# Iron deficiency anemia status in Iranian pregnant women and children: an umbrella systematic review and meta-analysis

**DOI:** 10.1186/s12884-024-06575-z

**Published:** 2024-05-22

**Authors:** Azadeh Dehghani, Roghayeh Molani-Gol, Maryam Rafraf, Fatemeh Mohammadi-Nasrabadi, Rahim Khodayari-Zarnaq

**Affiliations:** 1 Student Research Committee, Tabriz University of Medical Sciences, Tabriz, Iran; 2 Nutrition Research Center, Department of Community Nutrition, Faculty of Nutrition and Food Science, Tabriz University of Medical Sciences, Tabriz, Iran; 3 Research Department of Food and Nutrition Policy and Planning, Faculty of Nutrition Sciences and Food Technology, National Nutrition and Food Technology Research Institute, Shahid Beheshti University of Medical Sciences, Tehran, Iran; 4 Department of Health Policy and Management, School of Management and Medical Informatics, Tabriz University of Medical Sciences, Tabriz, Iran

**Keywords:** Prevalence, Iron deficiency anemia, Pregnant woman, Children, Systematic review, Meta-analysis

## Abstract

**Background:**

Iron deficiency anemia (IDA) is a global health challenge, especially affecting females and children. We aimed to conduct an umbrella systematic review of available evidence on IDA’s prevalence in Iranian pregnant women and children.

**Methods:**

We searched the Web of Science, Science Direct, PubMed, Scopus, and Google Scholar databases for articles published by April 2023. Meta-analyses investigating the status of IDA in Iran were included. The findings of seven meta-analyses comprising 189,627 pregnant women with a mean age of 26 and 5,890 children under six years old were included in this study. The methodological quality of each study was evaluated with the Assessment of Multiple Systematic Reviews (AMSTAR2) instrument.

**Results:**

We estimated the prevalence of IDA at 15.71% in pregnant women and 19.91% in young children. According to our subgroup analysis of pregnant women, IDA’s prevalence in urban and rural regions was 16.32% and 12.75%; in the eastern, western, central, southern, and northern regions of Iran, it was estimated at 17.8%, 7.97%, 19.97%, 13.45%, and 17.82%, respectively.

**Conclusion:**

IDA is common in young children and pregnant females and is a significant public health concern in Iran. The present umbrella review results estimated that Iran is in the mild level of IDA prevalence based on WHO classification. However, due to sanctions and high inflation in Iran, the prevalence of anemia is expected to increase in recent years. Multi-sectoral efforts are required to improve the iron status of these populations and reduce the burden of IDA in the country.

**Supplementary Information:**

The online version contains supplementary material available at 10.1186/s12884-024-06575-z.

## Introduction

Anemia is a global health challenge that affects human health and socioeconomic development. While it can affect any individual, its prevalence is higher among pregnant and pediatric populations [1]. The World Health Organization (WHO) states that anemia affects roughly five hundred million reproductive-age women globally. Among women aged 15 to 49, over a third of those who were pregnant (32.4 million) and almost a third of those who were not pregnant (496 million) were anemic in 2011. The maximum rates of anemia have been recorded in South Asia and West and Central Africa [2]. In the developing world, roughly 33% of children aged below 4 and 50% of those aged 5 to 15 have anemia [3].

Iron is vital for growth and metabolism, affecting the electron transport chain, oxidation-reduction, DNA replication, hormone synthesis, and reactive oxygen species (ROS) defense [4–6]. The lack of erythrocytes and hemoglobin in anemia impairs the ability of the body to deliver oxygen to vital organs via the blood [7].

Iron deficiency anemia (IDA) has many dangerous maternal and neonatal complications [8, 9]. IDA in pregnant women augments the risk of premature delivery, mortality, pre-eclampsia, maternal sepsis, and low birth weight of the child; it can also affect the cognitive development of the child [8–10]. Several factors, such as nutrition, genetics, frequent labor, abortions, multiparity, and infectious diseases, are related to anemia, but iron deficiency (ID) accounts for 75% of cases [10–12]. One of the main causes of ID is a gap between the body’s increased demand (up to seven times) for iron during pregnancy and the inadequate intake and low bioavailability of iron [13, 14].

An IDA prevalence above 5% indicates a public health problem in any country. IDA accounts for roughly half of all cases of anemia and is the most common nutritional deficiency disorder worldwide, affecting the health of millions of people [15]. The most vulnerable groups are young children and pregnant females [16]. In the developing world, IDA is prevalent in between 40 and 88% of women [17].

Proper and good monitoring of anemia in developing countries will be effective in planning for better control of this disease. The public health importance of anemia in terms of serum hemoglobin levels in a population can be determined using WHO criteria (40% or higher = Severe, 20.0-39.9% = Moderate, 5.0-19.9% = Mild, and 4.9% or lower = Normal) [18]. According to the previous studies in Iran, the prevalence rate varied from 10 to 30% [19, 20]. However, a comprehensive analysis was yet to be conducted.

According to the WHO report, a remarkable decrease in anemia has been achieved in some settings. However, the expected progress has been insufficient overall. In order to reach the WHO target, which is a 50% diminution in anemia in reproductive-age women by 2025, more and better actions are needed [2]. To address these matters and ameliorate the knowledge base for superior decision-making and future research, a general assessment of the prevalence of IDA among Iranians is essential, as data on this topic is limited or outdated. Hence, we aimed to conduct an umbrella systematic review of the available evidence on IDA prevalence in young children and pregnant women in Iran.

## Methods

### Search strategy

We conducted our systematic review and umbrella meta-analysis study as per the Preferred Reporting Items for Systematic Reviews and Meta-Analyses (PRISMA) framework [21] (Supplementary Table 1). This investigation was undertaken as a Ph.D. thesis titled “Policy analysis and development of upcoming policy options for the prevention of IDA in Iran,” approved by and registered with the Research Vice Chancellery of Tabriz University of Medical Sciences, Iran.

**Table 1:** PICOS criteria for inclusion of studies

Parameter	Inclusion criteria
Condition	Anemia
Context	Iran
Population	All age groups
Study design	Systematic Review Meta Published in English and Farsi dated up to April 2023

### Data sources and literature search

We searched PubMed, Google Scholar, Science Direct, Scopus, and Web of Science for all systematic reviews and meta-analyses on IDA in Iran published up until April 2023. We used the following search terms: iran OR Iran AND meta-analysis OR review, systematic OR systematic review AND anemia, iron deficiency OR hemoglobin OR iron OR “iron deficiency” OR iron deficiency anemia OR IDA OR ferritin OR iron-deficien* AND prevalence OR frequency OR percent OR mean OR estimation OR status.

Two authors conducted the literature search separately, with consensus being used to resolve disagreements. Furthermore, the “cited by,” “related articles,” and reference lists of the included studies were manually searched for further eligible studies. Supplementary Table 2 elucidates the search strategy.

**Table 2:** Characteristics of the included studies in Umbrella Systematic Review and Meta-Analysis of IDA prevalence in Iranian pregnant women and children

First author (year)	Number of primary studies; type of review	Time	residence place	Sample size	Age of participants	Participants	Anemia criteria Hb	IDA criteria Ferritin	Findings
Barooti et al., (2010) [16]	19, systematic review and meta-analysis	Between the years 1993 and 2007	urban in 12 studies, urban–rural in 3 studies, not stated 4 studies	11,037	NM	Pregnant Women	Hb < 11 g/dl Hb = 11 in ten studies, Hb = 10.5 in four studies, Hb = 12 in one study	Ferritin threshold level more than 15 ng/dl, normal ferritin level above 12 ng/dl, ferritin = 10 in one study, ferritin = 12 in three studies, ferritin = 15 in one study	The prevalence of anemia in pregnant women was 13.59% (95% CI:8.3–19.0).
Sayehmiri et al. (2015) [35]	32, systematic review and meta-analysis	1 January 1991 to 31 march 2015	Urban in 16 studies, urban-rural in 5 studies, rural in 2, not stated in 6 studies	63,372	Mean age 25.6	Pregnant Women	Hb < 11 g/dl Hb = 11 in 21 studies Hb = 10.5 in five studies	Ferritin cut-off point for IDA (ng/dl) Ferritin = 10 in one study, Ferritin = 12 in four studies, Ferritin = 15 in one study, Ferritin = 25 in one study	The prevalence of anemia among pregnant women is 14.2% (95% CI: 12-16.3).
Sayehmiri et al. (2015) [34]	12, systematic review and meta-analysis	1991 to 2015	NM	7087	NM	Pregnant women in different trimesters	below 11 g/dl in all studies Hb = 11 in 8 studies Hb = 10.5 in 2 studies	-	The prevalence of anemia in the first trimester of pregnancy was estimated at 19.6% (95% confidence interval, 8.4–30.9), in the second trimester 10.1% (95% confidence interval: 3.7–16.4), in the third trimester of pregnancy was estimated at 16.1% (95% confidence interval: 22-10.2).
Azami et al. (2016) [37]	18, systematic review and meta-analysis	January 1st, 2005 and December 31st, 2015	Urban in 5 studies, urban-rural in 3 studies, rural in 4, not stated in 6 studies	51,521	Mean age 26.17	Pregnant women in different trimesters	Below 11 g/dl in all studies	-	The prevalence of anemia in pregnant women was 17.9% (CI: 95%; 14.7–21.1).
Azami et al. (2016) [36]	25, systematic review and meta-analysis	March 2005 to 20 March 2016	Urban in 11 studies, urban-rural in 5 studies, rural in 4, not stated in 6 studies	56,610	Mean age 26.26	Pregnant women in different trimesters	Below 11 g/dl in all studies	-	The prevalence of anemia was 17% (95% CI: 14–20).
Akbari et al. (2017) [38]	27, systematic review and meta-analysis	December 1990 to 31 January 2016	Urban in 24 studies, urban–rural in two studies, and rural in one study	11 493	Less than 18 years	Children and adolescents	10.5 g/dl in two studies, below 11 g/dl six studies, below 11.5 g/dl three studies, below 12 g/dl 14 studies, and below 13 g/dl one study	Below 10 mg/l in four studies, below 12 mg/l in 13 studies, below 13.5 mg/l in one study, and below 15 mg/l in three studies	The prevalence of anemia was 13.9% (95% CI: 10.8–17.1) and the overall prevalence of ID was 26.9% (95% CI: 19.7–34.1). The prevalence of IDA was 7.9% (95% CI: 4.1–11.7) in males and 8.5% (95% CI: 6.1–10.8) among females aged under than 18 years.
Nazari et al. (2019) [39]	7, systematic review and meta-analysis	2001 to 2018	NM	1700	Under 6 years of age	Children	-	-	The prevalence of iron deficiency and IDA among the Iranian children under 6 years of age was 27.7% (95% CI: 11.9–43.5), and 18.2% (95% CI: 14.3–22), respectively.

CI: Confidence interval, IDA: Iron deficiency anemia, NM: Not mentioned

### Study screening and selection

Definition of keywords and screening of articles obtained from databases will be done according to PICO criteria (Table 1). For the present study, which is the type of prevalence studies, it is defined as CoCoPop, which includes the studied complication or disease (Condition), context or studied area (Context), and studied population (population) [22]. The search was unrestricted by the publication period. All English and Farsi publications performed in Iran until 2023 were eligible to include. The purpose of not having a time limit was to gain insight into the country’s current IDA situation. The inclusion criteria encompassed all meta-analyses reporting IDA prevalence in children and pregnant women in Iran with effect sizes (ES) and 95% confidence intervals (95% CIs). Two authors (AD and RMG) independently screened all publications against the eligibility criteria. In the first screening stage, the titles and abstracts were examined; the remaining articles were subsequently assessed for eligibility according to their full text. Disagreements were resolved by reaching a consensus. The data from the included studies were extracted and entered into an Excel spreadsheet containing a standardized data extraction form. The following data were extracted: primary author, year published, population, sex, number of participants and studies in the meta-analysis, anemia criteria, study type, effect sizes, and 95% CIs.

### Quality evaluation and evidence grading

We evaluated each included study using the Assessment of Multiple Systematic Reviews (AMSTAR2) instrument [23], a validated tool that examines the pooling method and study results through 16 questions. Each question is answered as yes, partial yes, no, or no meta-analysis. Then, each study is classified according to the final score as high, moderate, low, or very low quality. Based on Cochrane guidelines, we assessed the overall strength and quality of the studies using the GRADE tool. This tool assesses five risk factors of bias, consistency, directness, accuracy, and publication bias. Ultimately, the quality is graded in the same way as in the previous tool, declining if any of the mentioned factors are neglected [24].

### Data synthesis

We evaluated the pooled ES and 95% CI using random effects models with the restricted maximum likelihood approach. We examined for significant heterogeneity between studies using the I^2^ statistic and Cochrane’s Q test, indicated by I^2^ values above 50% or Q test p-values below 0.1 [25]. To identify sources of heterogeneity, we performed subgroup analysis based on sample size, sex, resident area, and region. Using sensitivity analysis, we examined how the overall ES would be affected if we removed a particular study. We performed Egger’s and Begg’s formal tests and examined the funnel plots visually to assess the impact of studies with at least 10 studies or less than 10 studies, respectively [26–28]. Using the “trim-and-fill” method, we corrected asymmetries observed as a result of the small study effect. P-values below 0.05 indicated significance. All analyses were done using STATA v. 17.0 (Stata Corporation, College Station, TX, USA).

## Results

### Literature search findings

**Fig. 1:**
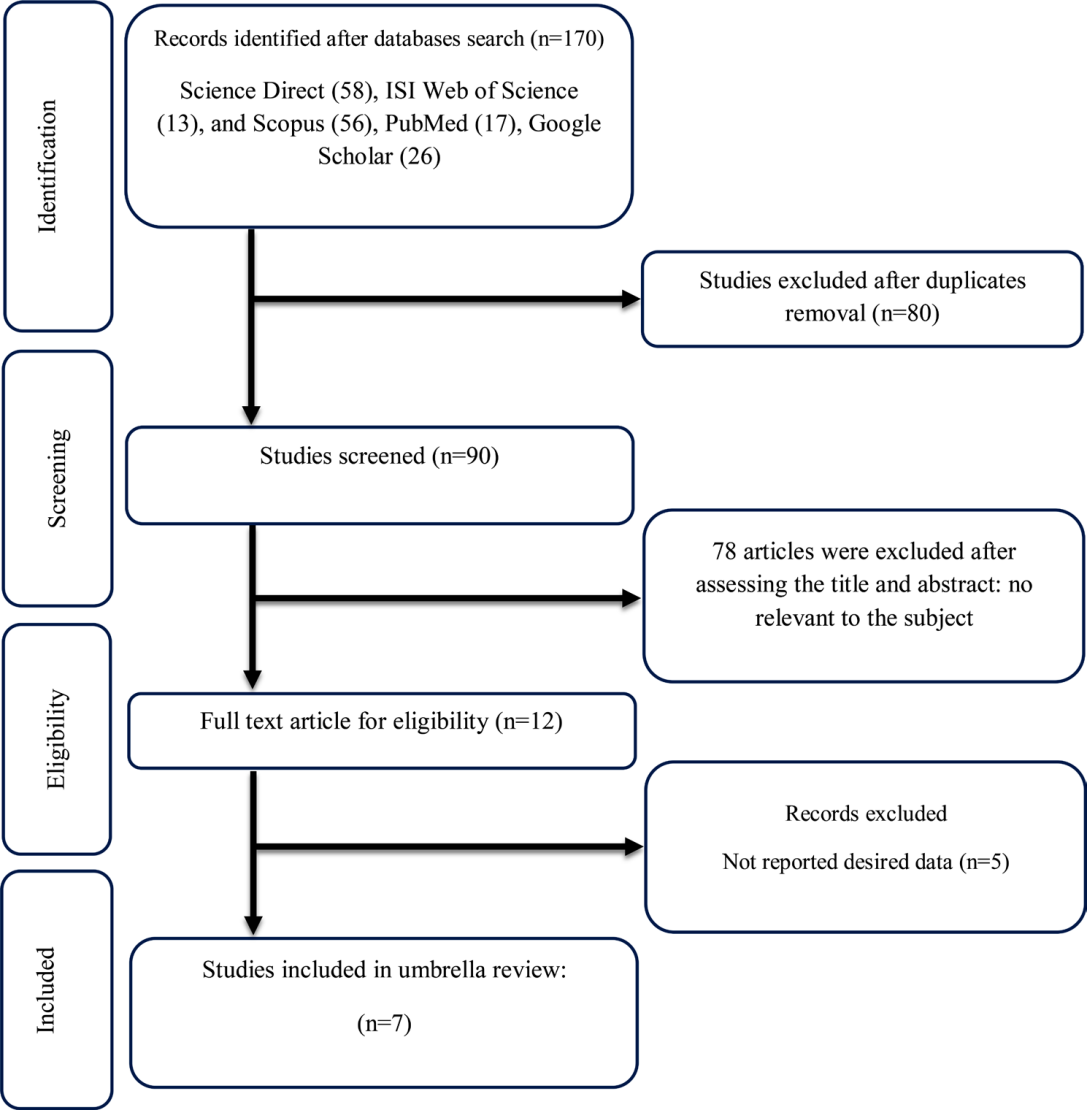
Flow chart of study selection for inclusion studies in the umbrella review

Figure 1 demonstrates the study flow diagram. We retrieved 170 publications from Science Direct (*n* = 58), Scopus (*n* = 56), Web of Sciences (*n* = 13), PubMed (*n* = 17), and Google Scholar (*n* = 26). We removed duplicates, leaving 90 articles for the title/abstract screening stage. After initial screening, 12 articles entered the full-text screening phase. During this phase, five articles were deemed to lack the required information for inclusion [29–33]. Finally, seven studies were deemed eligible for inclusion in our umbrella meta-analysis [16, 34–39].

### Study characteristics

The key characteristics of the reviewed articles are summarized in Table 2. All included studies were published by April 2023. A total of 189,627 pregnant women with a mean age of 26 years old and 5,890 children under 6 years (both genders) were included. The studies were conducted in different cities of Iran. In many studies, hemoglobin has been used to diagnose anemia, as well as ferritin as criteria for diagnosing IDA.

### Methodological quality

The results of our evaluation of the method of each study with the AMSTAR2 tool are presented in Table 3 [40]. We found that most publications (*n* = 6) were of moderate quality, with only one having high quality. Hence, the overall quality was moderate. Similarly, with the help of the GRADE instrument, all qualitative effects achieved a moderate rating (Table 4).

**Table 3:** Results of assessment of the methodological quality of included meta-analysis

Study	Q1	Q2	Q3	Q4	Q5	Q6	Q7	Q8	Q9	Q10	Q11	Q12	Q13	Q14	Q15	Q16	Quality assessment
Barooti, (2010)	Partial Yes	Yes	Yes	Partial Yes	Yes	No	Partial Yes	Yes	No	No	Yes	No	No	Yes	No	Yes	Moderate
Sayehmiri, (2015)	Partial Yes	Yes	Yes	Yes	Yes	Yes	Partial Yes	Yes	No	No	Yes	No	No	No	No	Yes	Moderate
Sayehmiri, (2015)	Partial Yes	Yes	Yes	Yes	Yes	Yes	Partial Yes	Yes	No	No	Yes	No	No	No	No	No	Moderate
Azami, (2016)	Partial Yes	Yes	Yes	Yes	Yes	Yes	Partial Yes	Yes	No	No	Yes	No	No	No	No	Yes	Moderate
Azami, (2016)	Partial Yes	Yes	Yes	Yes	Yes	Yes	Partial Yes	Yes	Yes	Yes	Yes	Yes	Yes	Yes	Yes	Yes	High
Akbari, (2017)	Partial Yes	Yes	Yes	Yes	Yes	Yes	Partial Yes	Yes	No	No	Yes	No	No	No	No	Yes	Moderate
Nazari, (2019)	Partial Yes	Yes	Yes	Yes	Yes	Yes	Partial Yes	Partial Yes	No	No	Yes	No	No	No	No	Yes	Moderate

* (1) Did the research questions and inclusion criteria for the review include the components of PICO? (2) Did the report of the review contain an explicit statement that the review methods were established prior to the conduct of the review and did the report justify any significant deviations from the protocol? (3) Did the review authors explain their selection of the study designs for inclusion in the review? (4) Did the review authors use a comprehensive literature search strategy? (5) Did the review authors perform study selection in duplicate? (6) Did the review authors perform data extraction in duplicate? (7) Did the review authors provide a list of excluded studies and justify the exclusions? (8) Did the review authors describe the included studies in adequate detail? (9) Did the review authors use a satisfactory technique for assessing the risk of bias (RoB) in individual studies that were included in the review? (10) Did the review authors report on the sources of funding for the studies included in the review? 11. If meta-analysis was performed, did the review authors use appropriate methods for statistical combination of results? 12. If meta-analysis was performed, did the review authors assess the potential impact of RoB in individual studies on the results of the meta-analysis or other evidence synthesis? 13. Did the review authors account for RoB in individual studies when interpreting/ discussing the results of the review? 14. Did the review authors provide a satisfactory explanation for, and discussion of, any heterogeneity observed in the results of the review? 15. If they performed quantitative synthesis, did the review authors carry out an adequate investigation of publication bias (small study bias) and discuss its likely impact on the results of the review? 16. Did the review authors report any potential sources of conflict of interest, including any funding they received for conducting the review?, Each question was answered with “Yes”, “Partial Yes” or “No”. When no meta-analysis was done, question 11, 12 and 15 were answered with “No meta-analysis conducted. Studies with ≥ 13 “yes” answers were categorized as “high”, 9–12 “yes” answers as “moderate”, 5–8 “yes” answers as “low”, and ≤ 4 “yes” answers as “critically low”

**Table 4:** Summary of results and quality of evidence assessment using the GRADE approach

Outcome measures	Summary of findings		Quality of evidence assessment (GRADE)					
	No. of patients/ number of meta-analyses (included studies)	Effect size (95% CI)	Risk of bias^a^	Inconsistency^b^	Indirectness^c^	Imprecision^d^	Publication bias^e^	Quality of evidence^f^
**Prevalence of IDA in pregnant woman**	189,627/5(105)	15.71 (13.987–17.435)	Not serious	Serious	Serious	Not serious	Not serious	Moderate
**Prevalence of IDA in under6 children**	5890/2(18)	19.91 (14.057–25.772)	Not serious	Not serious	serious	Not serious	Not serious	Moderate

^a^Risk of bias based on the AMSTAR results

^b^Downgraded if there was a substantial unexplained heterogeneity (I^2^ > 50%, *P* < 0.10) that was unexplained by meta-regression or subgroup analyses

^c^Downgraded if there were factors present relating to the participants, interventions, or outcomes that limited the generalizability of the results

^d^Downgraded if optimal information size was not met, or the 95%CI include the null value lower and upper bounds of the 95%CI were < 0.95 and > 1.05, respectively

^e^Downgraded if there was an evidence of publication bias using funnel plot

^f^Since all included studies were meta-analyses, the certainty of the evidence was graded as high for all outcomes by default and then downgraded based on prespecified criteria. Quality was graded as high, moderate, low, very low

### Iron deficiency anemia prevalence in pregnant women

**Fig. 2:**
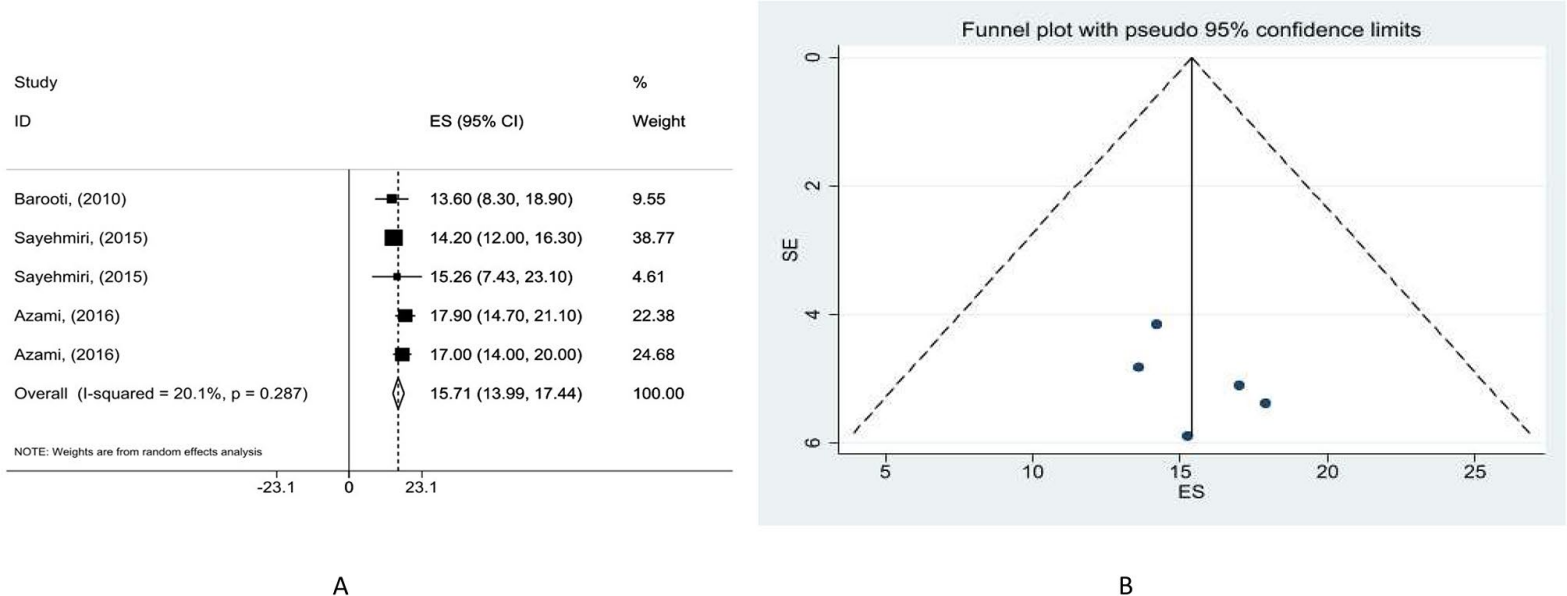
(**A**) Forest plot detailing effect sizes and 95% confidence intervals (CIs) and (**B**) funnel plot for the prevalence of IDA in pregnant woman

By combining the ES values of five meta-analyses of 105 studies comprising 189,627 pregnant women, we recorded an overall IDA prevalence of 15.71% (95% CI: 13.99, 17.44) (Fig. 2A). We also recorded low heterogeneity between the studies (I^2^ = 20.1%, *p* = 0.287).

Our subgroup analysis (Table 5) revealed that anemia was more prevalent in studies with sample size ≥ 2000 (ES = 16.12%, 95% CI: 13.766, 18.482) with a relatively high heterogenicity (I^2^ = 54.5%, *p* = 0.111) among the studies. The Begg’s tests indicated no small study effect (*p* = 0.327), with the funnel plot revealing no asymmetry (Fig. 2B). In addition, sub-group analysis based on area revealed that prevalence of anemia was 12.75% (95% CI: 4.89, 20.61) in rural regions, with low heterogeneity (I^2^ = 25.7%, *p* = 0.260) among the studies. Begg’s tests revealed no publication bias of significance (*p* = 0.117), and the funnel plot approved that too. In the urban areas, the prevalence of anemia was 16.32% (95% CI: 11.51, 21.14). Probably a high heterogeneity (I^2^ = 75.5%, *p* = 0.007) and no publication bias (*p* = 0.497) were observed. Also, in urban-rural and unknown areas the prevalence was (ES = 13.40%, 95% CI: 9.54, 17.27) and (ES = 15.27%, 95% CI: 12.29, 18.25), respectively. Heterogeneity was found to be low between the studies in both subgroups (I^2^ = 0.0%, *p* = 0.992). There was a significant publication bias (*p* = 0.042) in urban-rural and no significant publication bias in unknown areas (*p* = 0.602). However, after subgroup analysis based on location, we estimated the prevalence of anemia in the east (ES = 17.79%, 95% CI: 9.99, 25.58), west (ES = 7.97%, 95% CI: 5.04, 10.91), central (ES = 19.97%, 95% CI: 15.52, 24.43), south (ES = 13.45%, 95% CI: 8.76, 18.14) and north (ES = 17.82%, 95% CI: 15.43, 20.21). The studies were significantly heterogeneous in east (I^2^ = 83.4%, *p* = 0.002) and west (I^2^ = 67.0%, *p* = 0.048). We detected low heterogeneity in studies focused on the central (I^2^ = 0.0%, *p* = 0.687), south (I^2^ = 33.8%, *p* = 0.221), and north (I^2^ = 0.0%, *p* = 0.622) parts of Iran. No small study effect was noted in the east (*p* = 1.000), west (*p* = 0.602), center (*p* = 0.117), south (*p* = 0.221) and north (*p* = 0.602). Sensitivity analysis revealed no significant effect of excluding each particular study on the pooled ES among pregnant women (Supplementary Fig. 1).

### Iron deficiency anemia prevalence in young children

**Fig. 3:**
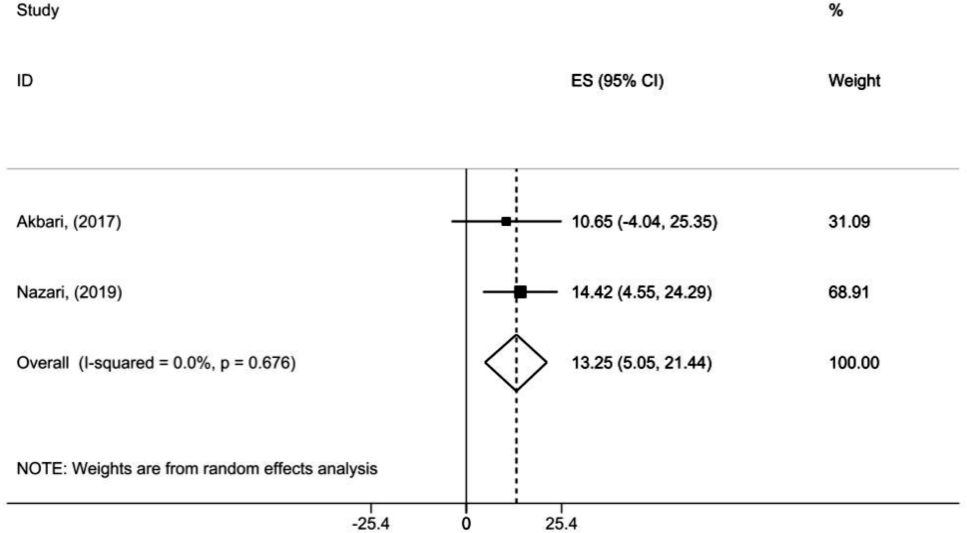
Forest plot detailing effect sizes and 95% confidence intervals (CIs) for the prevalence of IDA in all children under 6 years’ old

By combining two meta-analyses of 18 studies comprising 5,890 young children, we found an IDA prevalence of 19.91% (95% CI: 14.057, 25.772) (Fig. 3). Heterogeneity between studies was low (I^2^ = 0.0%, *p* = 0.743). Our subgroup analysis revealed an IDA prevalence of 14.75% of boys (95% CI: 5.933, 23.577) and 13.24% of girls (95% CI: 5.055, 21.441), with relatively low heterogenicity (I^2^ = 0.0%, *p* = 0.450 and I^2^ = 0.0%, *p* = 0.676, respectively) (Table 5).

**Table 5:** Subgroup analyses for the prevalence of iron deficiency anemia among Iranian pregnant woman and under 6 years’ child

	Effect size (*n*)	ES (95% CI) ^a^	*P*-within ^b^	I^2^ (%) ^c^	*P*-heterogeneity ^d^
Pregnant woman					
*Sample size*					
< 2000	2	14.121 (9.731, 18.511)	0.000	0.0	0.731
≥ 2000	3	16.124 (13.766, 18.482)	0.000	54.5	0.111
*Resident area*					
Rural	3	12.746(4.886, 20.606)	0.001	25.7	0.260
Urban	4	16.323(11.510, 21.137)	0.000	75.5	0.007
Rural-Urban	4	13.402(9.535, 17.268)	0.000	0.0	0.992
Unknown	3	15.267(12.285, 18.248)	0.000	0.0	0.922
*Region*					
East	3	17.785(9.995, 25.576)	0.000	83.4	0.002
West	3	7.972(5.036, 10.909)	0.000	67.0	0.048
Central	3	19.975(15.516, 24.433)	0.000	0.0	0.687
South	3	13.452(8.764, 18.140)	0.000	33.8	0.221
North	3	17.823(15.431, 20.215)	0.000	0.0	0.622
**Children**					
*sex*					
girls	2	13.248(5.055, 21.441)	0.002	0.0	0.676
boys	2	14.755(5.933, 23.577)	0.001	0.0	0.450

ES, Effect size; CI, confidence interval

^a^Obtained from the Random-effects model

^b^Refers to the mean (95% CI)

^c^Inconsistency, percentage of variation across studies due to heterogeneity

^d^Obtained from the Q-test

## Discussion

Our analysis indicates that IDA is prevalent in 15.71% of pregnant women and in 19.91% of children under 6 years old. Furthermore, the prevalence in pregnant women in urban areas is higher than in rural areas. This figure for the southern regions of Iran is lower than other geographical regions.

Our findings are consistent with studies conducted in Ethiopia (18%) [41], Pakistan (18.1%) [42] and Turkey (19.6%) [43], but the estimated rate in our country is lower than the results of studies recorded in Afghanistan (48.4%) [42], India (36.3%) [44], Côte d’Ivoire (20%) [45], Saudi Arabia (27%) [46] and Guatemala (30–60%) [47]. Also, in the United States, IDA is a concerning health challenge affecting 7% of females aged 12–49 years and up to 16% of pregnant women in this age range [48]. There are various programs to prevent iron deficiency anemia. But there are also obstacles about them, which can include the acceptance of these programs, insufficient priority, lack of awareness and training about its prevention and control; and the difficulty in meeting the needs of high-risk groups at certain times in their lives [11]. It seems that the prevalence of anemia is directly related to the socio-economic development of countries and the success of different countries in the prevention of anemia caused by iron deficiency. Global disparities and dissimilarity between countries and different exposure to multiple determinants of anemia indicate variation in the prevalence of anemia in them. Nationally representative demographic and health surveys in different low- and middle-income countries showed significant variation in the prevalence of anemia. At the national level, it has been shown that the prevalence of anemia has an inverse correlation with the economic development of countries [11]. Also, anemia is socially related to wealth, occupation (for example, agricultural workers), education and place of residence, which can justify the differences in various countries [49]. Moreover, malaria is one of the main causes of anemia in some countries that are malaria endemic areas, especially in Africa where there is a high prevalence of anemia. Therefore, malaria control can be one of the reasons for the difference in prevalence between countries [50].

IDA is a preventable widespread micronutrient deficiency [51], causing a significant health burden. Anemia, especially IDA, in pregnancy has been a neglected cause for a long time disability and is not considered as much as other diseases such as non-communicable diseases and cancer. However, it is time to look into it and understand that reducing IDA is just as impactful as other areas of focus [52]. Studies have shown that ID in women accounted for approximately 3% of global disability-adjusted life years (DALY) in 2010 [53]. Women’s health, especially reproductive health, indicates the nation’s health, which depends on various demographic and socio-economic factors [48]. It has been shown that that people aged 20 and 30 are more susceptible to anemia, encompassing many of the reproductive years [48]. Therefore, it is important for policymakers to determine the prevalence of anemia among women, especially in reproductive age.

Anemia caused by chronic ID leads to cognitive and behavioral disorders in infants and children, fatigue and reduced work ability in adolescents and adults, and perinatal mortality and premature births in pregnant females [54]. The results of statistics obtained from developing countries have shown that IDA is most prevalent in children younger than 5 years old. The most alarming range of its prevalence is 46.5% in Indonesia and between 33.7 and 50% in the African continent [38, 55, 56]. Anemia has been reported by different studies on Iranian teenagers and children at prevalence rates ranging from 9% to more than 40% [38, 57]. In the largest related study, the prevalence rate was 15% among Iranian children aged 2 to 12 [58]. Based on the WHO classification Iran was in the moderate range of IDA prevalence. However, according to our study, the prevalence of IDA is in the mild range [59]. WHO data is usually taken directly from the surveys conducted by the Ministry of Health and Medical Education of each country, which may not be part of the published documents and articles. While we used published meta-analysis studies in our umbrella meta-analysis. Due to the inflation and sanctions of recent years in Iran, the prevalence of anemia is expected to increase.

The National Integrated Micronutrient Survey I (NIMS-I) recently demonstrated that IDA is prevalent in all age/sex groups in Iran. These published results prompted a flour fortification program on the national scale, which aims to prevent deficiencies in iron and folic acid [60–63].

Iran is undergoing a nutritional transition and due to rapid socio-economic changes, lifestyle and food consumption have undergone changes. These changes in the consumption pattern in recent decades have greatly affected the micronutrient status of people [64]. In primary health care, prevention is the country’s priority instead of treatment. Despite the many programs and efforts of the government to eliminate malnutrition, the diet of the Iranian people in recent decades has been accompanied by less tendency towards healthy traditional diets and an increase in the tendency towards fast food options. Such an unhealthy diet can adversely impact the health of different social groups, especially children and women [65, 66].

Recent reports have shown that the amount of consumption of animal and vegetable products differs across urban and rural regions of Iran. For example, in Tehran city, the capital of Iran, a significant percentage of the energy intake is provided through the consumption of fatty foods and animal products. Animal products account for a minimum of 0.2% of energy intake in the southeast regions but exceed 11.0% in other provinces [66]. Sociocultural factors also influence food consumption in different regions [67].

The Iranian government is trying to adopt and implement appropriate programs in order to improve nutritional patterns as well as prevent obesity or underweight and cardiovascular diseases in metropolitan and rural regions and in all subgroups of populations [65, 66, 68]. Solutions like food fortification, supplementation, nutrition education and public health measures have been propounded as some crucial strategies to prevent and control micronutrient deficiency, including ID, by Iran’s Ministry of Health. In 1983, the government of Iran started the iron supplementation program. This program supplies free iron tablets to all women from the fifth month of pregnancy during regular visits to local health centers, continuing until three months postpartum [69]. Bread made from wheat flour undergoes iron and folic acid fortification and is subsidized by the government, particularly for large and low-income families. This option is low-cost and easily available in all regions [70].

Iron and folic acid supplements during pregnancy and after delivery diminish the risk of IDA and improve the consequences of childbirth [71–74]. Current WHO guidelines recommend that every pregnant woman in regions with a vast prevalence of anemia takes iron and folic acid supplements [75]. Many countries have implemented interventions to prevent anemia in young children and older girls since the 1970s [76, 77]. Interventions that are provided at the level of health centers do not operate on a large scale in most developing countries. This issue can be due to issues such as inappropriate provision of iron and folic acid supplements due to budget limitations, lack of demand from beneficiaries and health sectors, inefficient management of resources and stock outages [78–81]. The results of studies have shown that healthcare interventions and lifestyle modification in rural areas have had good results [16]. The observed difference in the prevalence of anemia in pregnant women between urban and rural regions is not statistically significant considering that their confidence intervals overlap with each other. This issue can be caused by the difference in the sample size, their lifestyle and diet, or the difference in the implementation of supplemental programs in the city and the village, as well as the difference in geographical areas [34, 36]. Poor nutritional behaviors and poor quality food products are potentially linked to anemia. In this regard, reducing the prevalence of anemia can be caused by more distribution of iron and folic acid supplements in rural health centers, as well as the program of flour fortification with iron and folic acid and consuming more bread in those areas [16, 82].

On the other hand, uncontrolled and unsupervised supplement consumption may be ineffective due to a lack of compliance or regularity [69]. Adherence to supplements is very different and influenced by demographic, social and health factors [83–88] and this reason can cause differences in different regions of Iran. According to reports, some of the key causes of anemia among Iranian children are factors related to nutrition and low iron intake [89].

Our findings align with Kadivar et al.’s study in Fars (southwest Iran) and Karimi et al.’s study in Yazd (central Iran). Similar to studies from different countries, we found no relationship between IDA and gender in young children [90–93]. It is also possible that these children have young mothers with frequent pregnancies, which cause depletion in iron stores, lower birth weight, IDA in the newborn and a higher probability of premature delivery [90]. However, because boys are born with less iron sources due to their higher weight at birth, and also because boys are exposed to more infections than girls, so male babies are at a greater risk of ID [94].

### Strengths and limitations

We conducted the first umbrella meta-analysis to summarize the results of contemporary studies on the prevalence of IDA in young children and pregnant women in Iran. We conducted the umbrella systematic review and meta-analysis on IDA prevalence in young Iranian children and pregnant women. However, some limitations exist. All included studies had cross-sectional meta-analysis designs, limiting our review given the observational nature. This means that it is not possible to obtain the cause-and-effect relationship from the results. The small number of subjects entered was another limitation. Also, the number of studies on children under six years old was very small. In addition, no study was found in adolescents and young people of reproductive age and puberty. The overlap of studies among meta-analyses increased the weight of studies that were included multiple times in separate meta-analyses. This can be accounted as a major limitation of our umbrella meta-analysis, which is unavoidable and may affect the overall result and confound the findings. Given the above, any interpretation must take this limitation into account. Moreover, using sensitivity analysis, we examined how the overall ES would be affected if we removed a particular meta-analysis study. Sensitivity analysis revealed no significant effect of excluding each study on the pooled ES. As recommended in the Cochrane Handbook [95], when there is a significant overlap, samples from meta-analyses are preferably identified based on individual data when available. Since the number of our studies was small, we preferred to maximize the sample size.

## Conclusion

The present umbrella review results estimated that Iran is in the mild level of IDA prevalence based on WHO classification. However, due to sanctions and high inflation in Iran, the prevalence of anemia is expected to increase in recent years. By understanding IDA’s prevalence in Iran, the authorities can improve strategies toward reaching the WHO target of a 50% diminution in anemia in reproductive-age women by 2025. Identifying local determinants and finding ways to improve the implementation of contextually appropriate strategies in IDA is very important to achieve global health goals. The causes of anemia are multifactorial, including infectious diseases, a complex interaction between nutrition, and other factors. This complexity leads to a challenge to effectively address the determining factors. Effective policy making and improving the implementation of appropriate strategies at the population level and reducing the knowledge gap in research will help reduce the burden of this disease in low-resource settings. Due to sanctions and high inflation in Iran, the prevalence of anemia is expected to increase in recent years. Our findings can improve evidence-informed decision-making, guide future research, and optimize programs aimed at preventing and controlling IDA in the most vulnerable population groups. Since systematic review and meta-analysis studies never replace national studies, it is recommended to design and implement a national study to estimate accurate statistics of the prevalence of anemia and its related factors in each geographical region of Iran.

### Electronic supplementary material

Below is the link to the electronic supplementary material.

Supplementary Material 1

Supplementary Material 2

Supplementary Material 3

## Data Availability

Data is provided within the manuscript or supplementary information files.
