# Supplementary material for: Iron deficiency anemia status in Iranian pregnant women and children: an umbrella systematic review and meta-analysis
Source: BMC Pregnancy Childbirth. 2024 May 22;24:381. doi: 10.1186/s12884-024-06575-z (PMC11110361; doi:10.1186/s12884-024-06575-z)
Supplement: Supplementary file 2 — Supplementary Material 2 [file 12884_2024_6575_MOESM2_ESM.docx]

**Supplementary Table 2.** The state of iron deficiency anemia among Iranian pregnant woman and child: an umbrella systematic review and meta-analysis reports: Method of the database search strategy using PubMed, Scopus, ScienceDirect, Google Scholar, and Web of Sciences

| **Database (Search**  **conducted up to**  **April, 2023)** | **Search terms^a^** | **Number of studies searched** |
| --- | --- | --- |
| PubMed | ((((iran[MeSH Terms]) OR (Iran[Title/Abstract])) AND (((Meta-analysis[Title/Abstract]) OR (review, systematic[MeSH Terms])) OR (systematic review[Title/Abstract]))) AND (((((((((anemia, iron deficiency[MeSH Terms]) OR (a, hemoglobin[MeSH Terms])) OR (Iron[Title/Abstract])) OR ("Iron deficiency"[Title/Abstract])) OR (anemia[Title/Abstract])) OR (IDA[Title/Abstract])) OR (Ferritin[Title/Abstract])) OR (hemoglobin[Title/Abstract])) OR (iron-deficien*[Title/Abstract]))) AND (((((((prevalence[MeSH Terms]) OR (frequency[Title/Abstract])) OR (percent[Title/Abstract])) OR (mean[Title/Abstract])) OR (estimation[Title/Abstract])) OR (status[Title/Abstract])) OR (prevalence[Title/Abstract])) | 17 |
| SCOPUS | ( TITLE-ABS-KEY ( iran ) ) AND ( ( TITLE-ABS-KEY ( meta-analysis ) OR TITLE-ABS-KEY ( systematic AND review ) ) ) AND ( ( TITLE-ABS-KEY ( iron ) OR TITLE-ABS-KEY ( anemia ) OR TITLE-ABS-KEY ( ida ) OR TITLE-ABS-KEY ( ferritin ) OR TITLE-ABS-KEY ( hemoglobin ) OR TITLE-ABS-KEY ( iron-deficient* ) ) ) AND ( ( TITLE-ABS-KEY ( prevalence ) OR TITLE-ABS-KEY ( frequency ) OR TITLE-ABS-KEY ( percent ) OR TITLE-ABS-KEY ( mean ) OR TITLE-ABS-KEY ( estimation ) OR TITLE-ABS-KEY ( status ) ) ) | 56 |
| Google Scholar | allintitle: anemia AND review AND Iran  allintitle: iron AND review AND Iran  allintitle: anemia AND systematic and Iran  allintitle: iron AND systematic and Iran  allintitle: anemia AND meta and Iran  allintitle: iron AND meta and Iran  Total | 5  5  4  3  4  5  26 |
| ScienceDirect | Title, abstract, keywords: anemia AND review AND Iran  Title, abstract, keywords: iron AND review AND Iran  Title, abstract, keywords: IDA AND review AND Iran  Title, abstract, keywords: anemia AND systematic AND Iran  Title, abstract, keywords: iron AND systematic AND Iran  Title, abstract, keywords: anemia AND meta AND Iran  Title, abstract, keywords: iron AND systematic AND Iran  Title, abstract, keywords: IDA AND meta AND Iran  Title, abstract, keywords: IDA AND systematic AND Iran  Total | 9  18  1  4  11  4  8  1  2  58 |
| Web of Sciences | #1 TS(Prevalence OR frequency OR percent OR mean OR estimation OR status) and Review Article (Document Types)  #2 TS=(Iron OR anemia OR IDA OR ferritin OR hemoglobin OR iron-deficient* ) and Review Article (Document Types)  #3 TS=(meta-analysis OR systematic review ) and Review Article (Document Types)  #4 TS=(Iran) and Review Article (Document Types)  #1 AND #2 AND #3 AND #4 | 350249  49097  259183  2591  13 |
| Total |  | 170 |

^a^ Searches were limited to original articles, and studies published in the English and Farsi language using the appropriate filters and/or search terms depending on the database.
