# Supplementary material for: Iron deficiency anemia status in Iranian pregnant women and children: an umbrella systematic review and meta-analysis
Source: BMC Pregnancy Childbirth. 2024 May 22;24:381. doi: 10.1186/s12884-024-06575-z (PMC11110361; doi:10.1186/s12884-024-06575-z)
Supplement: Supplementary file 3 — Supplementary Material 3 [file 12884_2024_6575_MOESM3_ESM.docx]

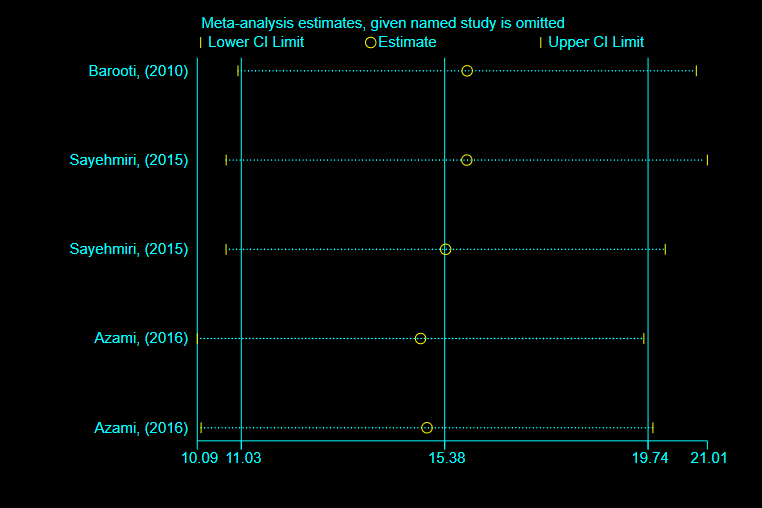


**Supp Figure 1.** The results of sensitivity analysis meta-analyses of the prevalence of anemia in pregnant women
